# The Cost-Effectiveness of Vaccination of Older Adults with an MF59-Adjuvanted Quadrivalent Influenza Vaccine Compared to Other Available Quadrivalent Vaccines in Germany

**DOI:** 10.3390/vaccines10091386

**Published:** 2022-08-25

**Authors:** Michele A. Kohli, Michael Maschio, Shannon Cartier, Joaquin Mould-Quevedo, Frank-Ulrich Fricke

**Affiliations:** 1 Quadrant Health Economics Inc., 92 Cottonwood Crescent, Cambridge, ON N1T 2J1, Canada; 2 Seqirus USA Inc., 25 Deforest Avenue, Summit, NJ 07901, USA; 3 Technische Hochschule Nürnberg, Bahnhofstraße 87, 90402 Nürnberg, Germany

**Keywords:** MF59-adjuvanted trivalent influenza vaccine, aTIV, high-dose trivalent influenza vaccine, TIV-HD, MF59-adjuvanted quadrivalent influenza vaccine, aQIV, cost-effectiveness, relative vaccine efficacy, rVE, cost saving

## Abstract

Enhanced quadrivalent influenza vaccines that include an adjuvant (aQIV) or a high dose of antigen (QIV-HD), which stimulate a stronger immune response in older adults than the standard vaccine (QIVe), are now approved. The objective of this research is to compare available vaccines and determine the cost-effectiveness of immunizing persons aged 65 years and above with aQIV compared to QIVe and QIV-HD in Germany. A compartmental transmission model calibrated to outpatient visits for influenza in Germany was used to predict the number of medically attended infections using the three vaccines. The rates of hospitalizations, deaths, and other economic consequences were estimated with a decision tree using German data where available. Based on meta-analysis, the rVE of −2.5% to 8.9% for aQIV versus QIV-HD, the vaccines are similar clinically, but aQIV is cost saving compared to QIV-HD (unit cost of EUR 40.55). All results were most sensitive to changes in vaccine effectiveness. aQIV may be cost-effective compared to QIVe depending on the willingness to pay for additional benefits in Germany. As aQIV and QIV-HD are similar in terms of effectiveness, aQIV is cost saving compared to QIV-HD at current unit prices.

## 1. Introduction

Individuals aged 65 years and above are at increased risk of complications and death from influenza [1,2,3,4]. In addition, these older adults have a lower immune response to vaccines than younger people [5]. Several countries have adopted the use of “enhanced” influenza vaccines that have been designed to boost the immune response in older adults. The MF59-adjuvanted trivalent influenza vaccine (aTIV), which elicits a stronger antibody response than conventional egg-based trivalent vaccines [6,7], was first approved in 1997 and has been used in older adults in more than 35 countries including Spain and Italy [8]. More recently, it has been recommended in the United Kingdom since the 2018/19 influenza season. A high-dose version of the trivalent vaccine (TIV-HD), which contains four times the amount of antigen than the standard version [9,10], has been available in countries such as the United States [11]. A recent meta-analysis has shown that both of these enhanced vaccines have greater effectiveness in persons aged 65 years and above compared to standard-dose trivalent influenza vaccine (TIVe) [12].

In 2017, the German Standing Committee on Vaccination (STIKO) at the Robert Koch Institute (RKI) recommended that all individuals aged 60 years and above be vaccinated annually with a quadrivalent influenza vaccine [13]. Until 2020, only standard egg-based quadrivalent influenza vaccines (QIVe) were available for vaccination of this age group in Germany. However, immunosenescence in these older adults has led to lower QIVe effectiveness [14]. Quadrivalent versions of the enhanced vaccines, including the MF59-adjuvanted version (aQIV) and the high-dose version (QIV-HD), are now available in Germany. STIKO preferentially recommended the use of QIV-HD in those aged 60 years and above [14,15] based on the results of a randomized controlled trial (RCT) comparing the trivalent version of the high-dose vaccine to the standard-dose trivalent vaccine [9]. While there are no RCTs comparing aTIV and TIVe, multiple studies of real-world effectiveness have been published [12,16]. Standard or adjuvanted influenza vaccines would not be covered by statutory health insurance (SHI), given the preferential recommendation for QIV-HD by STIKO and its adoption by the G-BA (Gemeinsamer Budesausschuss or Federal Joint Committee). However, in March 2021, the German Ministry of Health provided an executive directive that allows all quadrivalent vaccines, including aQIV and QIVe, to be prescribed and reimbursed for adults aged 60 years and above. The executive order has been prolonged in February 2022 and is currently valid until 31 March 2023 [17].

As multiple quadrivalent vaccines will be available in Germany for the 2022/23 seasons, this study aims to compare the potential clinical and economic impact of each of these. The specific research objective is to determine the cost effectiveness of vaccination of adults aged 65 years and above with aQIV compared to QIVe and QIV-HD in Germany.

## 2. Materials and Methods

### 2.1. Overview

In this analysis, the number of infections in the German population with various vaccination strategies was predicted using a transmission model, and then the economic consequences of those infections were calculated. The vaccine was QIVe, aQIV or QIV-HD for those in the target population aged 65 years and older. To understand the potential impact of the vaccines, we compared model runs where all those aged 65 years and over who received a vaccine were given QIVe (QIVe strategy) to one where all received aQIV (aQIV strategy). We also compared model runs where all those aged 65 years and over who received a vaccine were given QIV-HD (QIV-HD strategy) to the aQIV strategy. The base case analyses were conducted using the societal perspective, while sensitivity analyses were conducted using the SHI perspective [18]. The time horizon was 10 influenza seasons extracted from available data from the RKI [19,20,21,22,23,24,25,26,27] and the World Health Organization (WHO) [28] on the 2010/11 to 2019/20 seasons. A discount rate of 3.0% for both costs and economic outcomes (quality-adjusted life-years (QALYs)) was used, consistent with information from the Institute for Quality and Efficiency in Health Care (IQWiG) [29]. The results of most analyses are presented as average annual outcomes.

### 2.2. Model Structure

A compartmental transmission model with a susceptible–exposed–infectious–recovered (SEIR) structure was used to predict the number of influenza infections with and without vaccination. The model structure was similar to that of Baguelin and colleagues, who simulated independent influenza seasons to conduct cost-effectiveness analyses of vaccine policy changes for the United Kingdom [30,31,32,33,34]. The model structure is described in more detail in the Supplementary Material. The model was populated with two different types of seasons whose inputs were developed through a calibration process [35] that is described in the Supplementary Material, where rates of influenza infection were matched to rates of outpatient visits from an analysis of a German database [4]. One type of season represented the circulation of influenza A only, while the second represented circulation with both influenza A and B infections. All infections (symptomatic and asymptomatic) were included in the transmission model, but the main outcome of the model was infections seen by medical professionals.

### 2.3. Vaccine Effectiveness

The effectiveness of QIVe was estimated from a meta-analysis of test-negative design studies for standard vaccines set to 62%, 24% and 79% against A/H1N1, A/H3N2 and B types, respectively, for adults aged 65 years and older using assumptions described in the Supplementary Material [36]. As transmission models include the entire population, characteristics of vaccines used by age groups outside of the target population had to be specified. They were held constant for all strategies so they did not impact the comparison of interest. QIVe was used for persons aged 6 months to 64 years. Vaccine types and effectiveness values for the other age groups in the model are described in the Supplementary Material. Overall effectiveness against A and B types was calculated as a weighted average of the strains and therefore varied annually based on the proportion of strains circulating. The types of infections circulating for the 10 seasons of the analysis were derived from World Health Organization FluNet data for Germany based on the 2010/11 to 2019/20 seasons [28]. As shown in Table 1, years 1, 3, 6 and 8 were assumed to be years with both influenza A and B infections based on the 2010/11 to 2019/20 data. The proportion of influenza A infections caused by H1N1 from each of these 10 seasons was also used for the base case analysis (Table 1).

The relative vaccine effectiveness (rVE) of aTIV compared to TIVe [8,37,38,39,40] and aTIV compared to TIV-HD has been estimated in several observational cohort studies [37,38,39,41,42,43,44]. The relative vaccine effectiveness is defined as one minus the rate ratio of the two vaccines. The rate ratio is defined as the ratio of the incidence rates of the two vaccines [41]. Coleman et al. [12] conducted a systematic literature review and meta-analysis of studies on aTIV, including those comparing aTIV to TIVe and TIV-HD. They estimated a statistically significant rVE of aTIV compared to TIVe for reducing any medical encounter as 13.9% (95% confidence interval (CI): 4.2% to 23.5%). They also estimated that the rVE of aTIV compared to HD-TIV for reducing any medical encounter due to influenza and/or pneumonia was not significantly different at 3.2% (95% CI: −2.5% to 8.9%) using data from four studies [37,38,39,41]. In randomized controlled trials, the immune response in those given aQIV and QIV-HD was shown to be noninferior to those receiving aTIV and TIV-HD, respectively, for homologous influenza strains and superior for the additional B strain [45,46,47]. We therefore assumed that the rVE of the quadrivalent versions of the vaccine was the same as that of the trivalent versions of the vaccine.

### 2.4. Vaccine Coverage

Influenza vaccine coverage in Germany declined to approximately 35% in those aged 60 years and above by the 2016/17 season [48]. Due to the ongoing COVID-19 pandemic, the influenza vaccine coverage rates are expected to increase once again in Germany. For the base case analysis, we therefore assumed coverage in those aged 60 years and above would be 40% for all 10 years of the analysis. In sensitivity analyses, this input varied from 35% to 40%. Vaccine coverage for the remainder of the population is shown in Table 2 [49]. Vaccine coverage for those aged under 60 depends on whether a person is considered at high risk of complication from an influenza infection. This proportion was derived from a previously published German model of influenza transmission [50].

**Figure 1:**
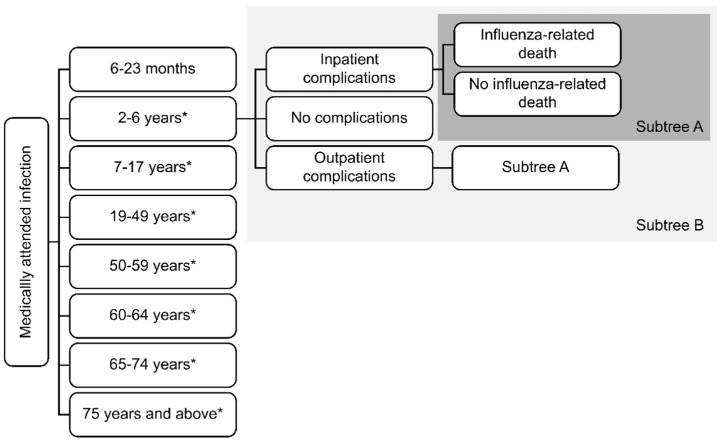
Resource use module structure. * The subtree B structure is repeated for all age groups, but this is not shown in the illustration.

The medically attended infections predicted by the dynamic model each season were entered into the probability tree shown in Figure 1 to determine the outcomes associated with infection. All individuals were assumed to receive, at minimum, outpatient treatment for their infection. Individuals who were asymptomatic or those who did not seek medical care for their symptoms did not enter the probability tree. Therefore, no economic consequences were assigned to symptomatic infections that were not seen for outpatient treatment. A portion of complicated cases received inpatient care, and then either died or survived. Hospitalization may have occurred for acute otitis media (AOM), community-acquired pneumonia (CAP) or severe influenza. A portion of patients that developed complications, including AOM and CAP, were treated on an outpatient basis. Those who developed CAP faced a probability of death.

### 2.5. Hospitalizations and Deaths

The proportion of cases that were hospitalized for each age group (Table 2) was based on the German database analysis used for the calibration [4]. Case fatality rates are not available for Germany. Therefore, German age-specific estimates of the mortality per population from the 2001/02 to 2013/14 seasons [53] were used to estimate the model inputs shown in Table 2 as described in the Supplementary Material. Mortality was assumed to occur only in individuals who were hospitalized or those aged 75 years and above who developed CAP but were treated in the community.

### 2.6. Severe Influenza Seasons

Over the past 10 years, Germany has experienced seasons where the morbidity associated with disease was higher than seen in our calibrated model. The average number of hospitalizations reported by the RKI for the 4 “severe” seasons indicated in Table 1 was 34,500. When we conducted a validation run with our model, as described in the Supplementary Materials, an average of only 13,936 hospitalizations were predicted. Furthermore, there was not one season where the predicted number of hospitalizations was above 21,500 (see Supplementary Materials Table S5). We therefore created the option to include severe seasons where the probability of experiencing a symptomatic infection that required medical care is double that in the original calibration. For the base case, it was assumed that 4 seasons were severe to be consistent with historical patterns. Scenario analyses were conducted where the number of severe seasons experienced was reduced to 2 and 0 seasons, as shown in Table 1.

### 2.7. Cost Inputs

The unit costs for the vaccines were based on the reimbursement price per dose. The average price of the multiple QIVe doses available was EUR 12.56, while aQIV was EUR 19.21, and QIV-HD was EUR 40.55 [54]. The cost of vaccine administration was EUR 7.95 for all vaccines based on standard fee schedules [55].

The cost of time lost from work was estimated using a human capital approach. The proportion of employed individuals aged 18 to 64 was estimated to be 74% [56]. Those individuals were estimated to lose EUR 160.04 in daily wages [57] for an average of 3.8 days [4] for each case of medically attended influenza. A sensitivity analysis was conducted using the friction cost approach whereby an elasticity of 80% was applied [29], reducing the average daily wage lost to EUR 128.03.

All other unit costs are displayed in Table 2. The unit costs of outpatient and hospital care were based on the German database analysis used to estimate rates of medical care [4]. The sickness benefit received for parental absenteeism to care for a sick child was estimated based on Dolk et al. [51] and included in the SHI perspective. As there were not sufficient data to update medical care unit costs using standard tariffs, these costs were inflated where required using the German consumer price index for health [57].

### 2.8. Utility Inputs

The reduction in QALYs, or the disutility weighted by time spent receiving health care, applied to uncomplicated cases of influenza by age is shown in Table 2 [51]. In addition, the reduction in AOM of 0.0001 for outpatient cases and 0.0034 for hospitalized cases was assumed for all ages [51]. An additional reduction in complications due to CAP was 0.0063 for outpatient cases and 0.0096 for inpatient cases [51]. Finally, all other hospitalizations for influenza received a reduction of 0.0047 [51]. The discounted number of QALYs lost due to death from influenza was calculated using expected survival [58] and utility values [52].

### 2.9. Analysis

The base case analysis was conducted from the societal perspective, assuming that 4 of the 10 seasons were severe, similar to the observed data for the past 10 seasons. Scenario analyses were conducted assuming 0 and 2 severe seasons for the societal and SHI cost perspectives, respectively. In addition, scenario analyses were completed using all combinations of the base case and rVE confidence interval limits for aQIV and QIV-HD from the meta-analysis. For those scenarios where the effectiveness of aQIV was lower than that of QIV-HD (rVE of aQIV compared to QIV-HD was −2.5%), the prices of QIV-HD required to achieve willingness-to-pay thresholds of EUR 20,000, EUR 30,000 and EUR 50,000 per QALY gained were calculated. While Germany does not have an explicit cost-per-QALY threshold, these thresholds have been cited by STIKO and used by other European decision makers [14,59,60].

Deterministic sensitivity analyses were conducted by varying vaccine effectiveness, vaccine coverage, case fatality rates, rates of hospitalization for influenza, and the QALY decrease for complications and no complications. Inputs for these sensitivity analyses are described above for vaccination coverage and vaccine effectiveness, while the remainder were based on 95% confidence intervals, which are displayed in the Supplementary Materials (Table S7). Finally, probabilistic sensitivity analyses were conducted for the base case scenario by varying inputs associated with the resource use probability tree, including the rate of hospitalizations, outpatient complications, mortality rates and utility decrements. A description of the inputs used is also provided in the Supplementary Materials (Table S7).

## 3. Results

The predicted number of influenza cases who received medical care, hospitalizations and deaths are displayed in Table 3 for the base case of four severe seasons along with the scenarios of two and zero severe seasons. Both of the enhanced vaccines reduced the number of influenza cases, hospitalizations and deaths in the German population compared to QIVe. As aQIV was the most effective vaccine in the base case, the use of this vaccine in the oldest age group resulted in the lowest amount of disease. The difference between the three available vaccines became more pronounced as the average number of severe seasons increased.

The costs by category, the total costs (societal perspective and SHI perspective) and the QALYs are shown in Table 4 for the base case of four severe seasons along with the scenarios of two and zero severe seasons. While the prevention of influenza does reduce the costs associated with treatment and productivity loss, these savings are not sufficient to fully offset the cost of vaccination. In all scenarios, QIVe was associated with the lowest costs, followed by aQIV and then by QIV-HD (highest costs). Therefore, for all scenarios, aQIV was compared to QIVe, and QIV-HD was compared to aQIV in the incremental analysis shown in Table 5. The incremental cost per QALY gained for aQIV compared to QIVe ranged from EUR 14,500 (four severe seasons) to EUR 23,000 (no severe seasons) for the societal cost perspective, while ratios for the SHI perspective ranged from EUR 17,000 to EUR 26,000, respectively. In all cases, aQIV dominated QIV-HD because it was considered marginally more effective in the base case (rVE = 3.2%), and it was less costly to implement.

For all eight effectiveness scenario analyses that are displayed in Table 6, QIVe was the least costly vaccination option, followed by aQIV and then by QIV-HD, which was the most expensive vaccination option. The total costs and QALYs associated with each of these scenarios are provided in the Supplementary Materials (Table S8). Once again, for all the incremental analyses in Table 6, aQIV was compared to QIVe, and QIV-HD was compared to aQIV. The incremental cost per QALY gained for aQIV compared to QIVe ranged from EUR 6700 to EUR 58,200 depending on the rVE of aQIV versus QIVe. QIV-HD was dominated by aQIV, except when the rVE of aQIV versus QIV-HD was −2.5%. In these scenarios, QIV-HD was more effective than aQIV, but given the price differential between the vaccines, the incremental cost per QALY gained was always above EUR 360,000 (Table 6). To further understand how vaccine price affects the incremental results, we conducted a threshold analysis where the price of QIV-HD was reduced so that the incremental cost per QALY was lower than several willingness-to-pay thresholds. If QIV-HD was more effective than aQIV (rVE: −2.5%), the price of QIV-HD needed to be in the range of EUR 20.31 to EUR 22.40 to be considered cost-effective at the thresholds considered (Supplementary Materials Table S7). If QIV-HD was considered to have the same or lower effectiveness than aQIV, then, by definition, the price must be lower than EUR 19.21.

**Figure 2:**
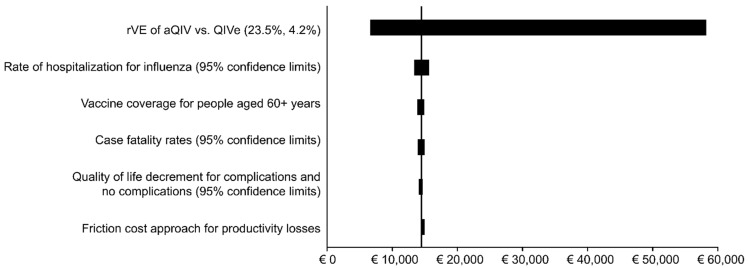
Tornado diagram: The impact of various deterministic sensitivity analyses on the estimated incremental cost-effectiveness ratio (societal perspective).

**Figure 3:**
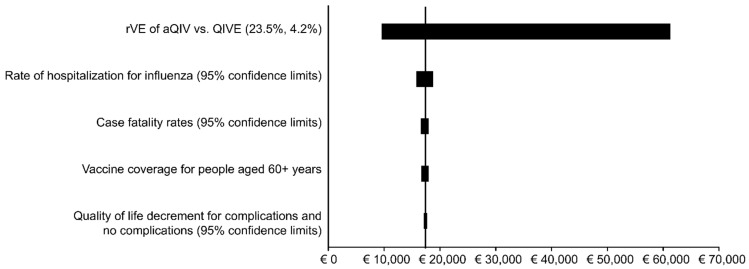
Tornado diagram: The impact of various deterministic sensitivity analyses on the estimated incremental cost-effectiveness ratio (SHI perspective).

The impact of all the deterministic sensitivity analyses for the comparison of aQIV with QIVe are shown in tornado diagrams in Figure 2 (societal perspective) and Figure 3 (SHI perspective). These diagrams illustrate that vaccine effectiveness has the greatest impact on the predicted cost effectiveness. This is followed by the rate of hospitalization, where the incremental cost-per-QALY ratio ranges from EUR 13,300 to EUR 15,700 for the societal perspective. Vaccination coverage, case fatality rates, the reduction in QALYs for complications and no complications and the use of the friction cost approach for productivity costs instead of the human capital approach have even smaller impacts on the ICER.

The cost-effectiveness acceptability curve comparing all vaccines using the base case effectiveness inputs is shown in the Supplemental Materials Figure S2. This shows that when the rVE of aQIV compared to QIVe is 13.9%, the probability that the vaccine will be cost-effective is 100% at a willingness-to-pay threshold of EUR 16,000. As aQIV is more effective than QIV-HD (an rVE of 3.2% of aQIV compared to QIVe), QIV-HD is dominant in all analyses and has a 0% probability of being cost effective.

## 4. Discussion

In this analysis, we demonstrated that enhanced vaccines with improved effectiveness, such as aQIV or QIV-HD, have the potential to prevent hospitalizations and deaths in those aged 65 years and above compared to QIVe. When the rVE of aQIV versus QIVe was 13.9% and the vaccine price was EUR 19.21 (or approximately 1.5 times the unit cost of QIVe), the incremental cost per QALY was EUR 14,500 under base case assumptions. As the meta-analysis used to inform the effectiveness inputs concluded that QIV-HD has similar effectiveness to aQIV, the clinical impact of QIV-HD and aQIV was projected to be similar. Given that the unit cost of QIV-HD (EUR 40.55) was approximately double that of aQIV, aQIV was always cost saving. The cost effectiveness of aQIV was most sensitive to vaccine effectiveness, followed by the rate of hospitalization due to influenza. Varying other model inputs had a less important impact on the results of this economic analysis.

Overall, the results of this analysis are consistent with a health economic analysis conducted for STIKO by adapting a previously published transmission model [61]. Based on the analysis, STIKO concluded that an enhanced vaccine could be good value for money for an older age group [14]. They found, for example, that an enhanced vaccine with an rVE of 15% compared to the standard QIVe could be double the cost of QIVe and still be cost effective considering a cost-per-QALY threshold of EUR 50,000.

While the quadrivalent version of the MF59-adjuvanted quadrivalent influenza vaccine has only recently been approved for use in Germany, the trivalent version of the vaccine has been used for several years in other countries. A systematic literature review of previous economic analyses in high-income countries concluded that the trivalent version of the adjuvanted vaccine was cost effective across a range of settings [62]. In several of the reviewed cost-effectiveness studies where aTIV was compared to the standard TIVe, aTIV dominated, as it was more effective and less costly than TIVe. As in our analysis, the results of the reviewed analyses were most sensitive to changes in the estimate of vaccine effectiveness.

One of the limitations of this study is that the effectiveness data that are available come from studies with trivalent versions of vaccines. Although immunogenicity data indicate that the quadrivalent versions are likely to perform similarly but with additional benefit for the extra influenza B type in the quadrivalent versions, the real-world effectiveness of the quadrivalent versions has yet to be determined. Currently, STIKO has preferentially recommended the use of QIV-HD based on the results of a randomized controlled trial [9], which showed that TIV-HD was significantly superior [14] to TIVe in people aged 65 years and over. While aTIV has been used in some regions for multiple years, there is no RCT comparing infection outcomes with aTIV and TIVe; therefore, STIKO has not yet recommended the use of aQIV over QIVe.

One further limitation is the lack of head-to-head comparisons of the effectiveness of the quadrivalent enhanced vaccines. The only available data comparing the various vaccines come from the United States, where trivalent vaccines (TIVe, TIV-HD, aTIV) and QIVe have been used for several seasons. As the US Advisory Committee on Immunization Practices does not provide a preferential recommendation for any of these vaccines, database analyses comparing the effectiveness of these vaccines in preventing medical encounter outcomes (including physician visits, hospitalizations, and emergency department visits) have therefore been possible. Coleman et al. [12] conducted a meta-analysis using the results from several studies from the 2017/18 and 2018/19 seasons in the United States to compare these four vaccines [37,38,39,41,42,43,44]. In addition, they identified two studies from Italy that compared aTIV to TIVe that were eligible for inclusion in the meta-analysis [8,40]. They concluded that aTIV was more effective than TIVe, with a 95% confidence interval for the rVE of 4.2% to 23.5%, but that aTIV was not significantly different from TIV-HD. Given these results, the cost-effectiveness of aQIV compared to QIVe does vary from EUR 6700 to EUR 58,200 in this analysis. Ultimately, data from multiple seasons comparing the use of the enhanced vaccines in the same populations will be required to determine whether either aQIV or QIV-HD provides superior protection against influenza.

Adverse events were not modelled in this analysis, as the costs are typically a minor contribution to the overall cost of vaccination because the rate of serious adverse events associated with vaccines are necessarily low [63,64,65]. STIKO has reported that additional local site adverse events may occur with aQIV and QIV-HD than with QIVe [14]. For aQIV compared to QIVe, the relative risk (RR) was 1.90 (95% CI: 1.50–2.39; moderate certainty of evidence). The RR of QIV-HD compared to QIVe was 1.40 (95% CI: 1.20–1.64; low certainty of evidence). The risk of systemic adverse events for aQIV was also reported to be higher than QIVe (RR: 1.18; 95% CI: 1.02–1.38; moderate certainty of evidence).

A final limitation of this analysis is the limited data on some of the outcomes associated with influenza in Germany, including the rate of symptomatic infection and mortality. To overcome this limitation, a calibration process was used to develop an influenza A-only season and an influenza A and B season and ensure that the model predicted cases of medically attended influenza reported in a German database analysis. The burden of influenza can vary greatly by season, and the severity of illness experienced can be an important driver of vaccine cost effectiveness. To increase the variability of influenza burden in this analysis, we created several severe season scenarios and additional sensitivity analyses with the rates of hospitalization and the case fatality rates. While the incremental cost per quality-adjusted life-year did increase in years when the influenza burden was lower, it remained below EUR 30,000 when comparing aQIV to QIVe using the base case rVE of 13.9%. While there is uncertainty in the annual burden of illness each year, in years with low severity, the adoption of an enhanced vaccine such as aQIV for persons 65 years and over remains good value for money.

## 5. Conclusions

In conclusion, enhanced vaccines such as aQIV or QIV-HD have the potential to prevent morbidity and mortality associated with influenza in Germany. This analysis demonstrated that aQIV may be cost effective compared to the standard QIVe depending on the willingness to pay for additional benefits given current clinical evidence. As aQIV and QIV-HD are similar in terms of effectiveness, aQIV is cost saving compared to QIV-HD at current unit prices.

## Figures and Tables

**Table 1:** Characteristics of the influenza seasons for the base case analysis.

Influenza Data			Model Runs				
Season	Percent of Infections Due to Type A (%) [28]	Percent of A That Was H1N1 (%) [28]	Year	Influenza Types Included	4 Severe Seasons	2 Severe Seasons	0 Severe Seasons
2010–2011	63.6	99.1	1	A and B	Normal	Normal	Normal
2011–2012	81.2	0.8	2	A Only	Normal	Normal	Normal
2012–2013 *	65.6	52.0	3	A and B	Severe	Normal	Normal
2013–2014	92.0	33.5	4	A Only	Normal	Normal	Normal
2014–2015 *	78.4	20.0	5	A Only	Severe	Severe	Normal
2015–2016	47.3	93.8	6	A and B	Normal	Normal	Normal
2016–2017 *	94.1	1.0	7	A Only	Severe	Severe	Normal
2017–2018 *	33.3	86.5	8	A and B	Severe	Normal	Normal
2018–2019	100	50.1	9	A Only	Normal	Normal	Normal
2019–2020	89.2	48.9	10	A Only	Normal	Normal	Normal

* The average number of hospitalizations for these seasons is higher than that for the other seasons at 34,500 (see Supplementary Material Table S5 for more details).

**Table 2:** Additional inputs for the base case analysis.

	Age Group							
	6–23 Months	2–6 Years	7–17 Years	18–49 Years	50–59 Years	60–64 Years	65 Years and above	75 Years and above
Population at high risk of complication if infected [50]								
Proportion	6.0%	6.0%	7.5%	14.2%	14.2%	47.1%	47.1%	47.1%
Vaccination coverage								
Low Risk	4.7%	4.7%	4.6%	17.2%	23.4%	40.0%	40.0%	40.0%
High Risk	9.3%	9.3%	9.2%	34.4%	46.8%	40.0%	40.0%	40.0%
Probability of hospitalization for medically attended cases [4]								
Percent	3.27%	1.98%	0.84%	0.45%	0.52%	1.65%	1.65%	1.65%
Case fatality rate *								
Low Risk	1.7%	2.4%	4.0%	4.0%	4.0%	11.2%	18.5%	18.5%
High Risk	1.7%	2.4%	4.0%	4.0%	4.0%	19.4%	42.9%	42.9%
Cost of hospital admission ** [4]								
Average	EUR 5596	EUR 2387	EUR 1924	EUR 2608	EUR 2927	EUR 3771	EUR 3771	EUR 3771
Cost of medical care visits [4]								
Average	EUR 60	EUR 56	EUR 63	EUR 64	EUR 64	EUR 78	EUR 78	EUR 78
Sickness benefit [51]								
Average	EUR 67	EUR 60	EUR 48	EUR 0	EUR 0	EUR 0	EUR 0	EUR 0
Baseline utility values [52]								
Low Risk	0.96	0.96	0.96	0.94	0.91	0.88	0.86	0.79
High Risk	0.94	0.94	0.94	0.91	0.82	0.82	0.79	0.71
Decrement in QALYS: Uncomplicated influenza [51]								
Decrement	0.0058	0.0058	0.0058	0.0068	0.0068	0.0068	0.0088	0.0088

* Case fatality rates were generated through calibration as described in the text and applied only to hospitalized cases and to cases of outpatient community-acquired pneumonia in those aged 75 years and above. ** The costs are averages that include outpatient treatment for those who were hospitalized and treatment of those with complications such as acute otitis media and community-acquired pneumonia.

**Table 3:** Base case results: predicted average number of annual influenza cases, hospitalizations and deaths when using QIVe, aQIV and QIV-HD in Germany by number of severe seasons.

	Four Severe Seasons		Two Severe Seasons		No Severe Seasons	
	Number	Decrease from QIVe	Number	Decrease from QIVe	Number	Decrease from QIVe
Medically attended influenza cases						
QIVe strategy	1,944,156		1,594,401		1,336,972	
aQIV strategy	1,899,373	44,782	1,555,646	38,755	1,305,876	31,096
QIV-HD strategy	1,908,456	35,699	1,563,504	30,897	1,312,183	24,789
Hospitalizations						
QIVe strategy	19,567		16,151		13,463	
aQIV strategy	19,020	547	15,675	476	13,084	379
QIV-HD strategy	19,131	436	15,771	380	13,161	302
Deaths						
QIVe strategy	6011		5064		4136	
aQIV strategy	5684	327	4776	287	3911	225
QIV-HD strategy	5750	261	4834	229	3956	180

aQIV: MF59-adjuvanted quadrivalent influenza vaccine; QIVe: conventional egg-based quadrivalent influenza vaccine; QIV-HD: high-dose quadrivalent influenza vaccine.

**Table 4:** Base case economic results: predicted average annual discounted costs and quality-adjusted life-years associated with influenza in Germany for aQIV, QIV-HD, and QIVe by number of severe seasons.

	Four Severe Seasons	Two Severe Seasons	No Severe Seasons
Vaccinations			
QIVe strategy	EUR 223,410,322	EUR 223,410,322	EUR 223,410,322
aQIV strategy	EUR 268,764,366	EUR 268,764,366	EUR 268,764,366
QIV-HD strategy	EUR 414,306,516	EUR 414,306,516	EUR 414,306,516
Vaccine administrations			
QIVe strategy	EUR 141,382,403	EUR 141,382,403	EUR 141,382,403
aQIV strategy	EUR 141,382,403	EUR 141,382,403	EUR 141,382,403
QIV-HD strategy	EUR 141,382,403	EUR 141,382,403	EUR 141,382,403
Outpatient care: medical care visits and prescription costs			
QIVe strategy	EUR 113,139,594	EUR 92,901,473	EUR 78,146,550
aQIV strategy	EUR 110,385,752	EUR 90,516,980	EUR 76,229,662
QIV-HD strategy	EUR 110,943,940	EUR 91,000,127	EUR 76,618,230
Outpatient care: sickness benefit			
QIVe strategy	EUR 26,320,030	EUR 21,206,398	EUR 18,158,628
aQIV strategy	EUR 25,929,864	EUR 20,876,252	EUR 17,884,947
QIV-HD strategy	EUR 26,009,444	EUR 20,943,578	EUR 17,940,770
Inpatient Care			
QIVe strategy	EUR 55,409,260	EUR 45,924,639	EUR 38,300,100
aQIV strategy	EUR 53,740,358	EUR 44,470,926	EUR 37,140,711
QIV-HD strategy	EUR 54,078,002	EUR 44,764,925	EUR 37,375,287
Productivity Costs			
QIVe strategy	EUR 424,739,096	EUR 348,489,533	EUR 293,712,048
aQIV strategy	EUR 417,828,417	EUR 342,577,962	EUR 288,867,890
QIV-HD strategy	EUR 419,236,918	EUR 343,782,606	EUR 289,855,238
Total statutory health insurance costs			
QIVe strategy	EUR 559,661,608	EUR 524,825,233	EUR 499,398,003
aQIV strategy	EUR 600,202,743	EUR 566,010,927	EUR 541,402,088
QIV-HD strategy	EUR 746,720,305	EUR 712,397,549	EUR 687,623,205
Total societal costs *			
QIVe strategy	EUR 958,080,674	EUR 852,108,368	EUR 774,951,422
aQIV strategy	EUR 992,101,295	EUR 887,712,636	EUR 812,385,031
QIV-HD strategy	EUR 1,139,947,779	EUR 1,035,236,577	EUR 959,537,674
Quality-adjusted life-years			
QIVe strategy	64,924,575	64,933,569	64,941,582
aQIV strategy	64,926,929	64,935,634	64,943,207
QIV-HD strategy	64,926,454	64,935,217	64,942,879

aQIV: MF59-adjuvanted quadrivalent influenza vaccine; QIVe: conventional egg-based quadrivalent influenza vaccine; QIV-HD: high-dose quadrivalent influenza vaccine. * The sickness benefit cost is not included in the total statutory health insurance but not the total societal costs.

**Table 5:** The cost effectiveness of aQIV compared to QIVe and QIV-HD compared to aQIV by perspective and number of severe seasons.

Number of Severe Seasons	aQIV Compared to QIVe			aQIV Compared to QIV-HD		
	Incremental Discounted Annual Costs	Incremental Discounted Annual QALYS	Incremental Cost per QALY Gained	Incremental Discounted Annual Costs	Incremental Discounted Annual QALYS	Incremental Cost per QALY Gained
Societal perspective						
4	EUR 34,020,622	2354	EUR 14,500	EUR 147,846,484	−475	Dominated *
2	EUR 35,604,268	2065	EUR 17,200	EUR 147,523,941	−417	Dominated *
0	EUR 37,433,609	1625	EUR 23,000	EUR 147,152,642	−328	Dominated *
SHI perspective						
4	EUR 40,541,135	2354	EUR 17,200	EUR 146,517,562	−475	Dominated *
2	EUR 41,185,694	2065	EUR 20,000	EUR 146,386,622	−417	Dominated *
0	EUR 42,004,085	1625	EUR 26,000	EUR 146,221,117	−328	Dominated *

* QIV-HD is inferior to aQIV.

**Table 6:** Effectiveness scenario analyses: The impact of varying relative vaccine effectiveness on the cost-effectiveness of the enhanced vaccines (societal perspective).

rVE (aQIV vs. QIVe) (%)	rVE (aQIV vs. QIV-HD) (%)	Incremental Cost per QALY Gained	
		aQIV vs. QIVe	aQIV vs. QIV-HD
4.2	−2.5	USD 58,200	USD 361,546
	3.2		Dominated *
	8.9		Dominated *
13.9	−2.5	USD 14,500	USD 413,157
	3.2		Dominated *
	8.9		Dominated *
23.5	−2.5	USD 6700	USD 477,483
	3.2		Dominated *
	8.9		Dominated *

* QIV-HD is inferior to aQIV.

## Data Availability

Not applicable.

## Supplementary Materials

The following supporting information can be downloaded at: https://www.mdpi.com/article/10.3390/vaccines10091386/s1.

## References

[1] An der Heiden M., Buchholz U. (2017). Estimation of influenza-attributable medically attended acute respiratory illness by influenza type/subtype and age, Germany, 2001/02–2014/15. Influenza Other Respir. Viruses.

[2] Ehlken B., Anastassopoulou A., Hain J., Schröder C., Wahle K. (2015). Cost for physician-diagnosed influenza and influenza-like illnesses on primary care level in Germany--results of a database analysis from May 2010 to April 2012. BMC Public Health.

[3] Haas J., Braun S., Wutzler P. (2016). Burden of influenza in Germany: A retrospective claims database analysis for the influenza season 2012/2013. Eur. J. Health Econ..

[4] Scholz S., Damm O., Schneider U., Ultsch B., Wichmann O., Greiner W. (2019). Epidemiology and cost of seasonal influenza in Germany—A claims data analysis. BMC Public Health.

[5] Haralambieva I.H., Painter S.D., Kennedy R.B., Ovsyannikova I.G., Lambert N.D., Goergen K.M., Oberg A.L., Poland G.A. (2015). The impact of immunosenescence on humoral immune response variation after influenza A/H1N1 vaccination in older subjects. PLoS ONE.

[6] Camilloni B., Basileo M., Di Martino A., Donatelli I., Iorio A.M. (2014). Antibody responses to intradermal or intramuscular MF59-adjuvanted influenza vaccines as evaluated in elderly institutionalized volunteers during a season of partial mismatching between vaccine and circulating A(H3N2) strains. Immun. Ageing.

[7] Camilloni B., Basileo M., Valente S., Nunzi E., Iorio A.M. (2015). Immunogenicity of intramuscular MF59-adjuvanted and intradermal administered influenza enhanced vaccines in subjects aged over 60: A literature review. Hum. Vaccines Immunother..

[8] Mannino S., Villa M., Apolone G., Weiss N.S., Groth N., Aquino I., Boldori L., Caramaschi F., Gattinoni A., Malchiodi G. (2012). Effectiveness of adjuvanted influenza vaccination in elderly subjects in northern Italy. Am. J. Epidemiol..

[9] DiazGranados C.A., Dunning A.J., Kimmel M., Kirby D., Treanor J., Collins A., Pollak R., Christoff J., Earl J., Landolfi V. (2014). Efficacy of high-dose versus standard-dose influenza vaccine in older adults. N. Engl. J. Med..

[10] Wilkinson K., Wei Y., Szwajcer A., Rabbani R., Zarychanski R., Abou-Setta A.M., Mahmud S.M. (2017). Efficacy and safety of high-dose influenza vaccine in elderly adults: A systematic review and meta-analysis. Vaccine.

[11] Grohskopf L.A., Alyanak E., Broder K.R., Blanton L.H., Fry A.M., Jernigan D.B., Atmar R.L. (2020). Prevention and control of seasonal influenza with vaccines: Recommendations of the advisory committee on immunization practices—United States, 2020–2021 influenza season. MMWR Recomm. Rep..

[12] Coleman B.L., Sanderson R., Haag M., McGovern I. (2021). Effectiveness of the MF59-adjuvanted trivalent or quadrivalent seasonal influenza vaccine among adults 65 years of age or older, a systematic review and meta-analysis. Influenza Other Respir. Viruses.

[13] Robert Koch-Institut (2017). Statement of the German Standing Committee on Vaccination at the RKI. Recommendations of the Standing Committee on Vaccination (STIKO) at the Robert Koch Institute—2017/2018.

[14] Robert Koch Institute (2021). Resolution and Scientific Justification of the German Standing Committee on Vaccination (STIKO) for Updating Influenza Vaccination Recommendation for Adults ≥ 60 Years of Age.

[15] Robert Koch-Institut (2021). STIKO: Aktualisierung der Influenza-Impfempfehlung fur Personen im Alter von ≥ 60 Jahren.

[16] Gärtner B.C., Weinke T., Wahle K., Kwetkat A., Beier D., Schmidt K.J., Schwarz T.F. (2022). Importance and value of adjuvanted influenza vaccine in the care of older adults from a European perspective—A systematic review of recently published literature on real-world data. Vaccine.

[17] Bundesministerium fur Gesundheit Verordnumng zum Anspruch auf Schutzimpfung Gegen Influenza un Masern. https://www.bundesgesundheitsministerium.de/fileadmin/Dateien/3_Downloads/Gesetze_und_Verordnungen/GuV/S/Schutzimpfung_gegen_Influenza_und_Masern_VO_BAnz_AT_11.03.2021_V2.pdf.

[18] Robert Koch Institute, STIKO (2016). Modelling Methods for Predicting Epdeimiological and Health Economic Effects of Vaccinations—Guidance for Analyses to be Presented to the German Standing Committee on Vaccination (STIKO).

[19] Robert Koch-Institut (2019). Bericht zur Epidemiologie der Influenza in Deutschland Saison 2018/19.

[20] Robert Koch-Institut (2018). Bericht zur Epidemiologie der Influenza in Deutschland Saison 2017/18.

[21] Robert Koch-Institut (2017). Bericht zur Epidemiologie der Influenza in Deutschland Saison 2016/17.

[22] Robert Koch-Institut (2016). Bericht zur Epidemiologie der Influenza in Deutschland Saison 2015/16.

[23] Robert Koch-Institut (2015). Bericht zur Epidemiologie der Influenza in Deutschland Saison 2014/15.

[24] Robert Koch-Institut (2014). Bericht zur Epidemiologie der Influenza in Deutschland Saison 2013/14.

[25] Robert Koch-Institut (2013). Bericht zur Epidemiologie der Influenza in Deutschland Saison 2012/13.

[26] Robert Koch-Institut (2012). Bericht zur Epidemiologie der Influenza in Deutschland Saison 2011/12.

[27] Robert Koch-Institut (2011). Bericht zur Epidemiologie der Influenza in Deutschland Saison 2010/11.

[28] World Health Organization FluNet. https://www.who.int/influenza/gisrs_laboratory/flunet/en/.

[29] IQWiG (2017). Institute for Quality and Efficiency in Health Care: General Methods Version 5.0.

[30] Baguelin M., Jit M., Miller E., Edmunds W.J. (2012). Health and economic impact of the seasonal influenza vaccination programme in England. Vaccine.

[31] Baguelin M., Flasche S., Camacho A., Demiris N., Miller E., Edmunds W.J. (2013). Assessing optimal target populations for influenza vaccination programmes: An evidence synthesis and modelling study. PLoS Med..

[32] Baguelin M., Camacho A., Flasche S., Edmunds W.J. (2015). Extending the elderly- and risk-group programme of vaccination against seasonal influenza in England and Wales: A cost-effectiveness study. BMC Med..

[33] Thorrington D., van Leeuwen E., Ramsay M., Pebody R., Baguelin M. (2017). Cost-effectiveness analysis of quadrivalent seasonal influenza vaccines in England. BMC Med..

[34] Thorrington D., van Leeuwen E., Ramsay M., Pebody R., Baguelin M. (2019). Assessing optimal use of the standard dose adjuvanted trivalent seasonal influenza vaccine in the elderly. Vaccine.

[35] Vanni T., Karnon J., Madan J., White R.G., Edmunds W.J., Foss A.M., Legood R. (2011). Calibrating models in economic evaluation: A seven-step approach. Pharmacoeconomics.

[36] Belongia E.A., Simpson M.D., King J.P., Sundaram M.E., Kelley N.S., Osterholm M.T., McLean H.Q. (2016). Variable influenza vaccine effectiveness by subtype: A systematic review and meta-analysis of test-negative design studies. Lancet Infect. Dis..

[37] Izurieta H.S., Chillarige Y., Kelman J., Wei Y., Lu Y., Xu W., Lu M., Pratt D., Wernecke M., MaCurdy T. (2020). Relative effectiveness of influenza vaccines among the United States elderly, 2018–2019. J. Infect. Dis..

[38] Pelton S.I., Divino V., Shah D., Mould-Quevedo J., DeKoven M., Krishnarajah G., Postma M.J. (2020). Evaluating the relative vaccine effectiveness of adjuvanted trivalent influenza vaccine compared to high-dose trivalent and other egg-based influenza vaccines among older adults in the US during the 2017-2018 influenza season. Vaccines.

[39] Boikos C., Fischer L., O’Brien D., Vasey J., Sylvester G., Mansi J. Relative effectiveness of aTIV versus TIVe, QIVe, and HD-TIV in preventing medical encounters during the 2017–18 and 2018–19 influenza season in the United States. Proceedings of the National Foundation for Infectious Diseases Annual Conference.

[40] Cocchio S., Gallo T., Del Zotto S., Clagnan E., Iob A., Furlan P., Fonzo M., Bertoncello C., Baldo V. (2020). Preventing the risk of hospitalization for respiratory complications of influenza among the elderly: Is there a better influenza vaccination strategy? A retrospective population study. Vaccines.

[41] Izurieta H.S., Chillarige Y., Kelman J., Wei Y., Lu Y., Xu W., Lu M., Pratt D., Chu S., Wernecke M. (2019). Relative effectiveness of cell-cultured and egg-based influenza vaccines among elderly persons in the United States, 2017–2018. J. Infect. Dis..

[42] Izurieta H.S., Lu M., Kelman J., Lu Y., Lindaas A., Loc J., Pratt D., Wei Y., Chillarige Y., Wernecke M. (2020). Comparative effectiveness of influenza vaccines among U.S. medicare beneficiaries ages 65 years and older during the 2019–20 season. Clin. Infect. Dis..

[43] Van Aalst R., Gravenstein S., Mor V., Mahmud S.M., Wilschut J., Postma M., Chit A. (2020). Comparative effectiveness of high dose versus adjuvanted influenza vaccine: A retrospective cohort study. Vaccine.

[44] Divino V., Krishnarajah G., Pelton S.I., Mould-Quevedo J., Anupindi V.R., DeKoven M., Postma M.J. (2020). A real-world study evaluating the relative vaccine effectiveness of a cell-based quadrivalent influenza vaccine compared to egg-based quadrivalent influenza vaccine in the US during the 2017-18 influenza season. Vaccine.

[45] Essink B., Fierro C., Rosen J., Figueroa A.L., Zhang B., Verhoeven C., Edelman J., Smolenov I. (2020). Immunogenicity and safety of MF59-adjuvanted quadrivalent influenza vaccine versus standard and alternate B strain MF59-adjuvanted trivalent influenza vaccines in older adults. Vaccine.

[46] Seqirus UK Limited (2018). Fluad. Summary of Product Characteristics.

[47] Sanofi Pasteur Quadrivalent Influenza Vaccine (Split Virion, Inactivated) High-Dose. Summary of Product Characteristics. https://www.hpra.ie/homepage/medicines/medicines-information/find-a-medicine/results/item?pano=PA2131/015/001&t=Quadrivalent%20Influenza%20Vaccine%20.

[48] Robert Koch-Institut (2019). Impfquoten bei Erwachsenen in Deutschland—Aktuelles aus der KV-Impfsurveillance und der Onlinebefragung von Krankenhauspersonal.

[49] Cai R., Gerlier L., Eichner M., Schwehm M., Rajaram S., Mould-Quevedo J., Lamotte M. (2021). Cost-effectiveness of the cell-based quadrivalent versus the standard egg-based quadrivalent influenza vaccine in Germany. J. Med. Econ..

[50] Eichner M., Schwehm M., Hain J., Uphoff H., Salzberger B., Knuf M., Schmidt-Ott R. (2014). 4Flu—An individual based simulation tool to study the effects of quadrivalent vaccination on seasonal influenza in Germany. BMC Infect. Dis..

[51] Dolk C., Eichner M., Welte R., Anastassopoulou A., van Bellinghen L.A., Poulsen Nautrup B., van Vlaenderen I., Schmidt-Ott R., Schwehm M., Postma M. (2016). Cost-Utility of Quadrivalent Versus Trivalent Influenza Vaccine in Germany, Using an Individual-Based Dynamic Transmission Model. Pharmacoeconomics.

[52] Ara R., Brazier J.E. (2011). Using health state utility values from the general population to approximate baselines in decision analytic models when condition-specific data are not available. Value Health.

[53] Iuliano A.D., Roguski K.M., Chang H.H., Muscatello D.J., Palekar R., Tempia S., Cohen C., Gran J.M., Schanzer D., Cowling B.J. (2018). Estimates of global seasonal influenza-associated respiratory mortality: A modelling study. Lancet.

[54] Kassenarztliche Vereinigung Westfalen-Lippe (2021). Verordnung von Grippeimpfstoffen im SSB fur die Saison 2021/2022.

[55] Bestellmengen-so Sind die Regionalen Regeln. https://www.aerztezeitung.de/Medizin/Impfstoffe-Honorar-Bestellmengen-so-sind-regionalen-Regeln-401818.html.

[56] Statistisches Bundesamt (Destatis) Persons in Paid Employment: Germany, Years, Extent of Employment, Sex. https://www-genesis.destatis.de/genesis/online?sequenz=tabelleErgebnis&selectionname=12211-0011&language=en#abreadcrumb.

[57] Statistisches Bundesamt (Destatis) Gross Earnings and Hours of Work. https://www.destatis.de/EN/Themes/Labour/Earnings/Earnings-Earnings-Differences/Tables/quaterly-earnings.html.

[58] Office for National Statistics (2018). National Life Tables: United Kingdom, 2015–17.

[59] Claxton K., Martin S., Soares M., Rice N., Spackman E., Hinde S., Devlin N., Smith P.C., Sculpher M. (2015). Methods for the estimation of the national institute for health and care excellence cost-effectiveness threshold. Health Technol. Assess..

[60] Department of Health & Social Care (2018). Cost-Effectiveness Methodology for Vaccination Programmes. Consultation on the Cost-Effectiveness Methodology for Vaccination Programmes and Procurement (CEMIPP) Report.

[61] Weidemann F., Remschmidt C., Buda S., Buchholz U., Ultsch B., Wichmann O. (2017). Is the impact of childhood influenza vaccination less than expected: A transmission modelling study. BMC Infect. Dis..

[62] Loperto I., Simonetti A., Nardone A., Triassi M. (2019). Use of adjuvanted trivalent influenza vaccine in older-age adults: A systematic review of economic evidence. Hum. Vaccines Immunother..

[63] Prosser L.A., Harpaz R., Rose A.M., Gebremariam A., Guo A., Ortega-Sanchez I.R., Zhou F., Dooling K. (2019). A Cost-Effectiveness Analysis of Vaccination for Prevention of Herpes Zoster and Related Complications: Input for National Recommendations. Ann. Intern. Med..

[64] Prosser L.A., Meltzer M.I., Fiore A., Epperson S., Bridges C.B., Hinrichsen V., Lieu T.A. (2011). Effects of adverse events on the projected population benefits and cost-effectiveness of using live attenuated influenza vaccine in children aged 6 months to 4 years. Arch. Pediatr. Adolesc. Med..

[65] Prosser L.A., O’Brien M.A., Molinari N.A., Hohman K.H., Nichol K.L., Messonnier M.L., Lieu T.A. (2008). Non-traditional settings for influenza vaccination of adults: Costs and cost effectiveness. Pharmacoeconomics.

