# Supplementary material for: The Cost-Effectiveness of Vaccination of Older Adults with an MF59-Adjuvanted Quadrivalent Influenza Vaccine Compared to Other Available Quadrivalent Vaccines in Germany
Source: Vaccines (Basel). 2022 Aug 25;10(9):1386. doi: 10.3390/vaccines10091386 (PMC9503029; doi:10.3390/vaccines10091386)

Technical Appendix and Supplementary Material

The cost-effectiveness of vaccination of older adults with an MF59 adjuvanted quadrivalent influenza vaccine compared to other available quadrivalent vaccines in Germany

Authors: Kohli M, Maschio M, Cartier S, Mould-Quevedo J, Frank-Ulrich Fricke.

Table of Contents

1. Model Structure 3
2. Vaccine Effectiveness 4
3. Dynamic Model Calibration........ 5
4. Estimation of Case Fatality Rates 9
5. Validation of Model 10
6. Sensitivity Analyses Inputs 12
7. Additional Results Tables and Figures 14
8. References 18

List of Tables

Table S1. Details of vaccine effectiveness values. 5

Table S2. Ranges used for the calibration of the dynamic model for susceptibility and percent of cases that require medical attention. 6

Table S3. Final values for the calibration of the dynamic model for susceptibility and percent of cases that require medical attention. 7

Table S4. Targets and final predicted number of deaths by age group following calibration of the case fatality rates. 9

Table S5. Number of hospitalizations predicted by the model for all normal, all severe and mixed seasons assuming coverage as in Eichner 2014. 11

Table S6. Inputs for the probabilistic sensitivity analyses and selected deterministic sensitivity analyses. 12

Table S7. Threshold analysis: price required for QIV-HD in order for the incremental cost-per quality-adjusted life-year ratio to fall below various willingness-to-pay thresholds. 14

Table S8. Details results of effectiveness scenario analyses: The impact of varying relative vaccine effectiveness on the cost-effectiveness of the enhanced vaccines (societal perspective). 15

List of Figures

Figure S1. Transmission model structure 4
Figure S2 Target and final model predicted incidence of medically attended cases by age group for the A only and A&B scenarios. 8
Figure S3. Cost-effectiveness acceptability curve showing the probability that aQIV and QIV-HD are cost-effective compared to the current QIVe in Germany over a range of willingness-to-pay values. 16
Figure S4. Scatterplot of incremental costs and QALYs from the PSA simulation comparing aQIV to the current QIVe in Germany. 17
Figure S5. Scatterplot of incremental costs and QALYs from the PSA simulation comparing QIV-HD to aQIV in Germany. 17

1. Model Structure

The compartmental transmission model structure is shown in Figure S1. Each of the compartments is divided into 15 age groups and by risk of complication from infection (low or high). All individuals the simulation begin in the susceptible, unvaccinated compartment at the start of the influenza season, except for a few individuals representing the imported influenza that is required to start the epidemic. As in a standard SEIR (Susceptible, Exposed, Infected, Resistant) model, individuals move from susceptible and exposed according to the force of infection, which is a function of the rates of effective contacts between susceptible and unsusceptible individuals in the population. As with other transmission models,[1-4] the rate of effective contact is a function of the age-specific contact matrix[5] and the transmissibility of the virus per contact. Unique to this approach, as each season is treated independently, a proportion of the population in the susceptible compartment may actually have naturally acquired immunity from infection in the past season.

Following an effective contact, individuals develop a latent infection that cannot be transmitted and transition to the exposed compartment. Next, they move into the infection compartment where they can transmit their infection until entering the recovered compartment. As in Baguelin 2012[6], there are two exposed and two infected compartments (not shown in Figure S1) which allow the latent and infectious periods to be gamma-distributed. The rate of loss of latency is a function of the average latent period. Similarly, the loss of infectiousness is a function of the average duration of an influenza infection. Following recovery from an infection, individuals move into a recovered compartment and remain there until the end of the influenza season. At the start of a new season, the simulation is reset and all individuals are placed in the susceptible compartment again.

All model compartments are also stratified between individuals at low and high risk of complications from influenza (also not shown on Figure S1). Probability of transitioning between compartments is the same for low and high-risk individuals, and random mixing between the groups is assumed. These populations are separated to track differential vaccine coverage as well as differential risk of hospitalization and death following infection.

Influenza A is modelled independently from influenza B, using the same model structure. There is no vaccine cross-protection of A and B. Vaccine effectiveness is a function of the proportion of A/H1N1 and A/H3N2 infections and effectiveness against each A type. For type B infections, an overall effectiveness was applied to both Yamagata and Victoria lineages. Vaccine-mediated protection is assumed to last for 1 year (i.e., the entire influenza season). Vaccination is assumed

to occur at a constant rate between week 41 (October) and week 50 (December). Individuals in all unvaccinated compartments are eligible, as a portion of the infections would have occurred without development of clinical symptoms such that infection status may not be known by the individual. Effective vaccination is assumed to fully protect a portion the vaccinated individuals, while those unsuccessfully vaccination remain susceptible.

The time step of the simulation was set to be 0.1 days based on a range of epidemiological systems explored by Keeling and colleagues.[7] The model was programmed in Microsoft Excel.

Figure S1. Transmission model structure

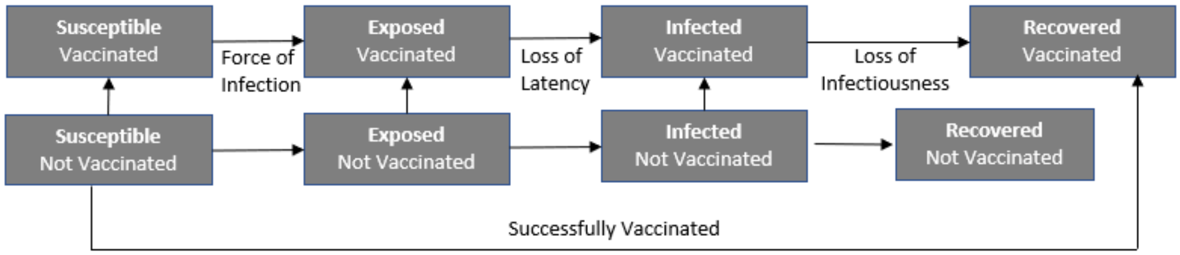

2. Vaccine Effectiveness

The age and strain-specific effectiveness of the egg-based quadrivalent influenza vaccine (QIVe) was estimated based on a systematic review of test-negative design studies of influenza vaccination conducted by Belongia and colleagues.[8] Several assumptions were made in order to use these data. First, Belongia did not limit the review to a particular type of vaccine, so it was assumed that the presented data applied to inactivated egg-grown vaccines as this would have been the most common vaccine used during the study period. Unlike other reviews,[9,10] adjustments were made to the B efficacy using data from Tricco and colleagues[9] because the vaccines reviewed by Belongia would have been primarily trivalent formulations. On average, trivalent formulations are less effective than quadrivalent formulations because they contain one B strain, which may or may not have matched the local circulating strain. Values for all ages are provided in Table S1.

Table S1. Details of vaccine effectiveness values.

Vaccine	Age Group (Years)	QIVe
A/H1N1*	Under 18	0.69
	18 to 64	0.73
	65 and above	0.62
A/H3N2*	Under 18	0.43
	18 to 64	0.35
	65 and above	0.24
B**	Under 18	0.70
	18 to 64	0.68
	65 and above	0.79

*Source: Belongia 2016[8]
**As Belongia 2016[8] presented an overall efficacy for B with TIVe, the relative difference between B matched and mismatched from the systematic review by Tricco (2013)[8] was used to calculate effectiveness for B type matched and mismatched.

3. Dynamic Model Calibration

During the calibration process one scenario that represents seasons with influenza A only and a second one to represent seasons with both A and B influenza infections were created.

The calibration targets were the age-specific cumulative incidence of infection that come to medical attention across one influenza season in a vaccinated population. The A only scenario was calibrated to the average medically attended incidence of influenza for 2012 and 2014 from an analysis of a German database.[11] The A&B scenario was calibrated so that the overall incidence matched the medically attended incidence of influenza for 2013 from this database analysis.[11] The proportion of infections attributed to B was assumed to be 47.5%, which was the average of the proportion of B infections during years that B accounted for more than 25% of cases (See Main Manuscript Table 1).

Several parameters were fixed based on the literature or previous cost-effectiveness analyses. The number of people in each of the 15 age-groups in the dynamic model was based on 2019 data from the Federal Statistical Office of Germany.[12] The population was distributed into low and high risk groups based on data from a previous analysis (Main manuscript Table 2)[13], but random mixing between the two groups was assumed. Vaccine coverage was based on Eichner 2014[13] as this represented the approximate coverage during the time period of interest (2012-2014). Vaccine effectiveness was set to be the same value for all seasons in the calibration. The type of influenza infection will impact the effectiveness of vaccination. For the calibration, the average

effectiveness of QIVe against influenza across 10 years was calculated using the type distribution shown in **Error! Reference source not found.** and the type specific efficacy for QIVe described in the main article. The average latent period was 0.77 days and the average duration of infectiousness was 1.59 days.[6] Transmissibility was estimated so that R_0, or the basic reproductive number, which was calculated using the simulation method described by Vynnycky and White,[14] was 1.6 as in previously published German models[13,15] It was assumed that 100 infections in each risk group seeded or started the influenza infection.

The susceptibility to infection and percent of infected cases who develop clinical symptoms and seek medical care were varied during the calibration process. One thousand combinations of these inputs were created from uniform distributions with ranges for each of the age groups shown in Table S2 using Latin hypercube sampling.[16] The model was run with each parameter set and the one that produced the best goodness of fit (using a least square measure) to the calibration targets was chosen.[16] The final calibrated input parameters are shown in Table S3. The results of the calibration, including the age-specific incidence of medically attended infections for each scenario are shown in Figure S2.

Table S2. Ranges used for the calibration of the dynamic model for susceptibility and percent of cases that require medical attention.

Age Group	Susceptibility	Symptoms*
0-1 yrs	0.5 - 1.0	0.07 - 0.21
2-6 yrs	0.5 - 1.0	0.07 - 0.21
7-17 yrs	0.5 - 0.9	0.07 - 0.14
18-24 yrs	0.5 - 0.9	0.07 - 0.14
25-29 yrs	0.5 - 0.9	0.07 - 0.14
30-34 yrs	0.5 - 0.9	0.07 - 0.14
35-39 yrs	0.5 - 0.9	0.07 - 0.14
40-44 yrs	0.5 - 0.9	0.07 - 0.14
45-49 yrs	0.5 - 0.9	0.07 - 0.14
50-54 yrs	0.5 - 0.9	0.07 - 0.14
55-59 yrs	0.5 - 0.9	0.07 - 0.14
60-64 yrs	0.5 - 0.9	0.07 - 0.14
65-69 yrs	0.5 - 0.9	0.07 - 0.14
70-74 yrs	0.5 - 0.9	0.07 - 0.14
75+ yrs	0.5 - 0.9	0.07 - 0.14

*Symptoms = Probability of developing symptoms and seeking medical care. The ranges shown are the same for those aged 7 and above, however, for susceptibility, each of the age groups in this table were calibrated independently. To simplify the calibration the age groups with similar shading were set to have equivalent probability of developing symptoms and seeking medical care.

Table S3. Final values for the calibration of the dynamic model for susceptibility and percent of cases that require medical attention.

Age Group	Type A		Type B	
	Susceptibility	Symptoms*	Susceptibility	Symptoms*
0-1 yrs	0.726	0.21	0.788	0.52
2-6 yrs	0.922	0.18	0.796	0.45
7-17 yrs	0.711	0.09	0.888	0.24
18-24 yrs	0.683	0.09	0.573	0.22
25-29 yrs	0.838	0.09	0.857	0.23
30-34 yrs	0.676	0.09	0.752	0.23
35-39 yrs	0.806	0.09	0.849	0.23
40-44 yrs	0.899	0.09	0.717	0.23
45-49 yrs	0.621	0.09	0.854	0.22
50-54 yrs	0.596	0.09	0.627	0.22
55-59 yrs	0.657	0.09	0.742	0.22
60-64 yrs	0.869	0.09	0.52	0.22
65-69 yrs	0.808	0.14	0.653	0.35
70-74 yrs	0.578	0.14	0.728	0.35
75+ yrs	0.712	0.14	0.814	0.35

* Symptoms = Probability of developing symptoms and seeking medical care

Figure S2 Target and final model predicted incidence of medically attended cases by age group for the A only and A&B scenarios.

Panel A: Influenza A (Results are the same for influenza A in both the A only and A&B scenarios).

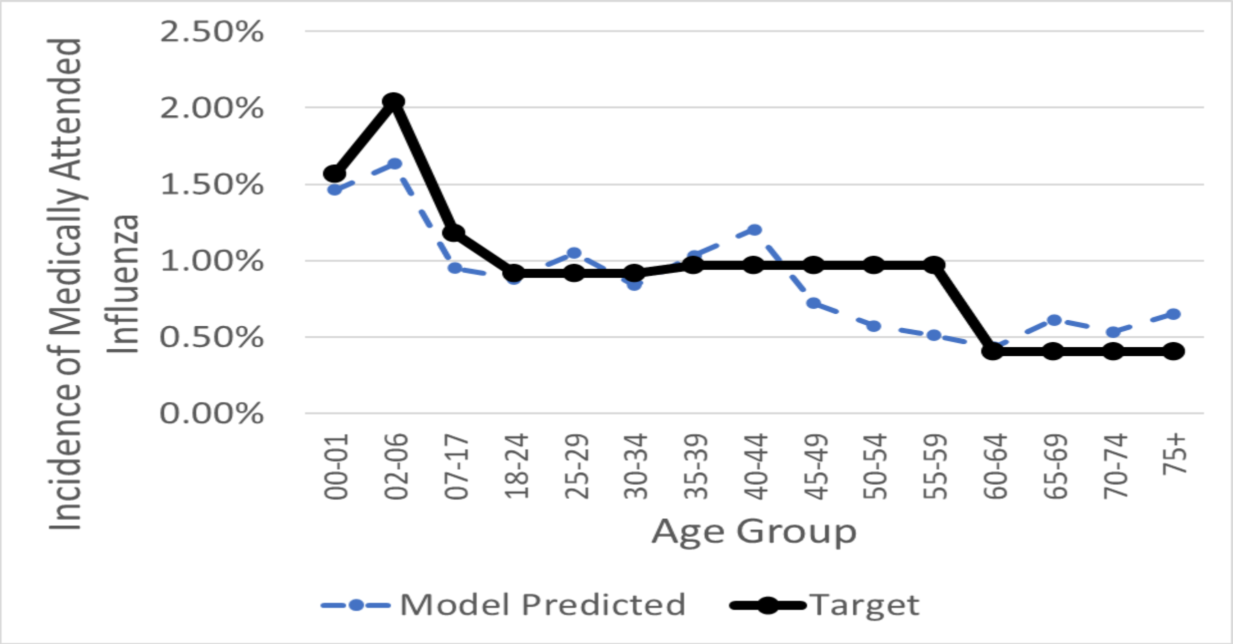

Panel B: Influenza B

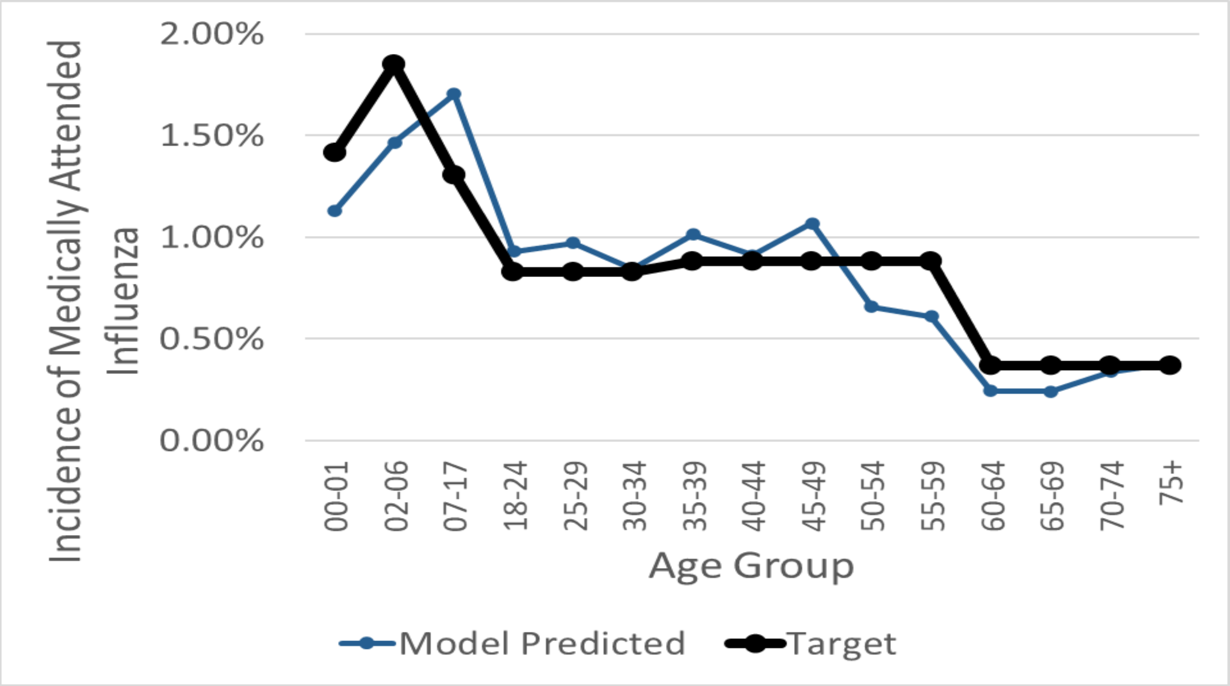

4. Estimation of Case Fatality Rates

Case fatality rates are not available for Germany. However, age-specific estimates of the mortality per population from the 2001/02 to 2013/14 seasons[17] were used to estimate these. The model was run for 10 years, assuming a constant coverage rate based on Eichner 2014[13] as this represents coverage during the season from which mortality targets were estimated. Case fatality rates based on UK data served as a starting point for the calibration process.[18] Case fatality rates were adjusted until the model predicted deaths for one season matched the targets generated using German data from Iuliano 2018[17] as shown in Table S4. The number of predicted deaths represents a conservative scenario when compared to estimates of excess deaths due to influenza in Germany as published by Zucs 2005.[19] They calculated a crude annual average number of deaths between 1985 and 2001 as 13,601 per year ranging from 3,670 to 33,234 depending on the year. When taking a more conservative approach, they estimated an annual average of 6,906 with a range of 60 to 21,365.

Table S4. Targets and final predicted number of deaths by age group following calibration of the case fatality rates.

Age group	German Population 2019-12-31	Mean annual influenza mortality rates (per 100,000 population)*	Calibration Target: Estimated Mean Number of Deaths	Calibration Results: Predicted Mean Number of Deaths**
6 - 23 months	1,192,500	0.4	5	17
2 -6 years	3,889,000	0.4	16	30
7-17 years	8,154,000	0.4	33	28
18-49 years	32,367,000	0.4	129	78
50-59 years	13,464,000	0.4	54	17
60-64 years	5,654,000	0.4	23	81
65-74 years	8,548,000	2.9	248	338
75 years +	9,539,000	21	2,003	2,161
**Total**	**82,807,500**		**2,510**	**2,751**

*For the calibration, case fatality inputs were adjusted until the model predicted deaths for one season matched the targets generated using data from Iuliano 2018.[17]

** To conduct the calibration, vaccine coverage was set as in Eichner 2014[13] for all years as this represents coverage during the seasons from which mortality targets were estimated, assuming all normal seasons.

5. Validation of Model

To conduct the model validation run, vaccine coverage in 2010/11 was set to values from Eichner 2014 and reduced linearly to coverage levels consistent with reports from the Robert Koch Institute (RKI) by 2016/17. Three runs were conducted as shown in Table S5. In one run, all seasons were set to be normal. In the second run, all seasons were set to be severe. In the final run, the historical data was used to designate 4 seasons to be severe. This final scenario with 4 seasons set to be severe was used for the base case analysis.

Table S5. Number of hospitalizations predicted by the model for all normal, all severe and mixed seasons assuming coverage as in Eichner 2014.

Year		Influenza Type	Proportion of A that is A/H1N1[20]	Hospitalizations Estimated by the Robert Koch Institute			Model Predicted (Normal Seasons)*	Model Predicted (Severe Seasons)*	Model Predicted (Mixed Normal & Severe)
				Estimate	95% Confidence Interval	Data Source			
1	2010/11	A & B	99.1%	4,700	2,800 – 6,600	[21]	7,800	15,600	7,800
2	2011/12	A Only	0.8%	7,400	5,500 - 9,200	[22]	12,614	25,227	12,614
3	2012/13*	A & B	52.0%	32,000	28,000 – 35,000	[23]	16,024	32,047	32,047+
4	2013/14	A Only	33.5%	3,100	1,700 – 4,500	[24]	10,730	21,461	10,730
5	2014/15*	A Only	20.0%	31,000	26,000 – 35,000	[25]	12,839	25,678	25,678+
6	2015/16	A & B	93.8%	16,000	13,000 – 19,000	[26]	18,184	36,369	18,184
7	2016/17*	A Only	1.0%	30,000	27,000 – 32,000	[27]	16,022	32,044	32,044+
8	2017/18*	A & B	86.5%	45,000	42,000 – 47,000	[28]	21,009	42,018	42,018+
9	2018/19	A Only	50.1%	18,000	16,000 – 20,000	[29]	11,996	23,992	11,996
10	2019/20	A Only	48.9%				12,145	24,290	12,145
**Average**				**20,800**			**13,936**	**27,873**	**20,526**

* To conduct these runs, vaccine coverage in 2010/11 was set as in Eichner 2014 and reduced linearly reduced to be consistent with reports on vaccine coverage from RKI.
+ Season is set to severe in the mixed normal and severe model results based on the number of hospitalizations from annual epidemiological summaries

6. Sensitivity Analyses Inputs

Table S6. Inputs for the probabilistic sensitivity analyses and selected deterministic sensitivity analyses.

Parameter Description		Base Case Value	Probabilistic Sensitivity Analyses		Deterministic Sensitivity Analyses	
Parameter	Age Group		SE Value	Distribution	Low Value	High Value
Hospitalization	6-23 months	0.0327	0.0033	Beta	0.0266	0.0394
	2-6 yrs	0.0198	0.0020	Beta	0.0161	0.0239
	7-17 yrs	0.0084	0.0008	Beta	0.0068	0.0101
	18-49 yrs	0.0045	0.0005	Beta	0.0037	0.0055
	50-59 yrs	0.0052	0.0005	Beta	0.0042	0.0062
	60-64 yrs	0.0165	0.0017	Beta	0.0135	0.0199
	65-74 yrs	0.0165	0.0017	Beta	0.0135	0.0199
	75 + yrs	0.0165	0.0017	Beta	0.0135	0.0199
Outpatient complications	6-23 months	0.2343	0.0117	Beta	0.2117	0.2576
	2-6 yrs	0.1562	0.0078	Beta	0.1412	0.1718
	7-17 yrs	0.0863	0.0043	Beta	0.0781	0.0950
	18-49 yrs	0.0503	0.0025	Beta	0.0455	0.0553
	50-59 yrs	0.0554	0.0028	Beta	0.0501	0.0610
	60-64 yrs	0.0994	0.0050	Beta	0.0899	0.1094
	65-74 yrs	0.0994	0.0050	Beta	0.0899	0.1094
	75 + yrs	0.0994	0.0050	Beta	0.0899	0.1094
Influenza-related case fatality rate (high risk)	6-23 months	0.0175	0.0009	Beta	0.0158	0.0192
	2-6 yrs	0.0175	0.0009	Beta	0.0158	0.0192
	7-17 yrs	0.0244	0.0012	Beta	0.0221	0.0269
	18-49 yrs	0.0400	0.0020	Beta	0.0361	0.0440
	50-59 yrs	0.0400	0.0020	Beta	0.0361	0.0440
	60-64 yrs	0.1940	0.0097	Beta	0.1753	0.2134
	65-74 yrs	0.4285	0.0214	Beta	0.3868	0.4708
	75 + yrs	0.4285	0.0214	Beta	0.3868	0.4708
Influenza-related case fatality rate (low risk)	6-23 months	0.0175	0.0009	Beta	0.0158	0.0192
	2-6 yrs	0.0175	0.0009	Beta	0.0158	0.0192
	7-17 yrs	0.0244	0.0012	Beta	0.0221	0.0269
	18-49 yrs	0.0400	0.0020	Beta	0.0361	0.0440
	50-59 yrs	0.0400	0.0020	Beta	0.0361	0.0440
	60-64 yrs	0.1120	0.0056	Beta	0.1013	0.1232
	65-74 yrs	0.1853	0.0093	Beta	0.1675	0.2038
	75 + yrs	0.1853	0.0093	Beta	0.1675	0.2038

QALYs lost for infections with complications	6-23 months	0.0034	0.0003	Beta	0.0028	0.0041
	2-6 yrs	0.0034	0.0003	Beta	0.0028	0.0041
	7-17 yrs	0.0034	0.0003	Beta	0.0028	0.0041
	18-49 yrs	0.0034	0.0003	Beta	0.0028	0.0041
	50-59 yrs	0.0034	0.0003	Beta	0.0028	0.0041
	60-64 yrs	0.0034	0.0003	Beta	0.0028	0.0041
	65-74 yrs	0.0034	0.0003	Beta	0.0028	0.0041
	75 + yrs	0.0034	0.0003	Beta	0.0028	0.0041
QALYs lost for infections, no complications,	6-23 months	0.0058	0.0006	Beta	0.0047	0.0070
	2-6 yrs	0.0058	0.0006	Beta	0.0047	0.0070
	7-17 yrs	0.0058	0.0006	Beta	0.0047	0.0070
	18-49 yrs	0.0068	0.0007	Beta	0.0055	0.0081
	50-59 yrs	0.0068	0.0007	Beta	0.0055	0.0081
	60-64 yrs	0.0068	0.0007	Beta	0.0055	0.0081
	65-74 yrs	0.0088	0.0009	Beta	0.0071	0.0106
	75 + yrs	0.0088	0.0009	Beta	0.0071	0.0106

QALY – quality-adjusted life years; yrs - years

7. Additional Results Tables and Figures

Table S7. Threshold analysis: price required for QIV-HD in order for the incremental cost-per quality-adjusted life-year ratio to fall below various willingness-to-pay thresholds.

rVE (aQIV vs QIV)		rVE (aQIV vs QIV-HD)*		€ aQIV	€ QIV-HD	WTP
Lower 95% CI	rVE=4.2%	Lower 95% CI	rVE=-2.5%	€ 19.21	€ 22.40	€ 50,000
					€ 21.24	€ 30,000
					€ 20.65	€ 20,000
Base	rVE=13.9%	Lower 95% CI	rVE=-2.5%	€ 19.21	€ 22.01	€ 50,000
					€ 20.99	€ 30,000
					€ 20.48	€ 20,000
Upper 95% CI	rVE=23.5%	Lower 95% CI	rVE=-2.5%	€ 19.21	€ 21.64	€ 50,000
					€ 20.76	€ 30,000
					€ 20.31	€ 20,000

aQIV - MF59 adjuvanted quadrivalent influenza vaccine; CI – Confidence Interval; QIVe – Conventional egg-based quadrivalent influenza vaccine; QIV-HD – High dose quadrivalent influenza vaccine; rVE – relative vaccine effectiveness; vs. – versus.

* This analysis was completed for the one scenario where QIV-HD is more effective than aQIV . Whenever QIV-HD is less effective than aQIV, QIV-HD must be priced less than aQIV or it will be dominated.

Table S8. Details results of effectiveness scenario analyses: The impact of varying relative vaccine effectiveness on the cost-effectiveness of the enhanced vaccines (societal perspective).

rVE (aQIV vs QIVe)		rVE (aQIV vs QIV-HD)		Strategy	Costs	QALYs	Incremental Costs	Incremental QALYs	ICER	Comparison
LCL	rVE= 4.2%	LCL	rVE=-2.5%	Current Strategy	€ 958,080,674	64,924,575	--	--	Ref	-
				aQIV (Age 65+)	€ 999,988,937	64,925,296	€ 41,908,263	720	€ 58,186	Current Strategy
				QIV-HD (Age 65+)	€ 1,143,622,106	64,925,693	€ 143,633,169	397	€ 361,546	aQIV (Age 65+)
LCL	rVE= 4.2%	Base	rVE=3.2%	Current Strategy	€ 958,080,674	64,924,575	--	--	Ref	-
				aQIV (Age 65+)	€ 999,988,937	64,925,296	€ 41,908,263	720	€ 58,186	Current Strategy
				QIV-HD (Age 65+)	€ 1,148,127,603	64,924,753	€ 148,138,666	-542	Dominated	-
LCL	rVE= 4.2%	UCL	rVE=8.9%	Current Strategy	€ 958,080,674	64,924,575	--	--	Ref	-
				aQIV (Age 65+)	€ 999,988,937	64,925,296	€ 41,908,263	720	€ 58,186	Current Strategy
				QIV-HD (Age 65+)	€ 1,153,235,027	64,923,680	€ 153,246,091	-1,616	Dominated	-
Base	rVE= 13.9%	LCL	rVE=-2.5%	Current Strategy	€ 958,080,674	64,924,575	--	--	Ref	-
				aQIV (Age 65+)	€ 992,101,295	64,926,929	€ 34,020,622	2,354	€ 14,454	Current Strategy
				QIV-HD (Age 65+)	€ 1,135,948,671	64,927,277	€ 143,847,376	348	€ 413,157	aQIV (Age 65+)
Base	rVE= 13.9%	Base	rVE=3.2%	Current Strategy	€ 958,080,674	64,924,575	--	--	Ref	-
				aQIV (Age 65+)	€ 992,101,295	64,926,929	€ 34,020,622	2,354	€ 14,454	Current Strategy
				QIV-HD (Age 65+)	€ 1,139,947,779	64,926,454	€ 147,846,484	-475	Dominated	-
Base	rVE= 13.9%	UCL	rVE=8.9%	Current Strategy	€ 958,080,674	64,924,575	--	--	Ref	-
				aQIV (Age 65+)	€ 992,101,295	64,926,929	€ 34,020,622	2,354	€ 14,454	Current Strategy
				QIV-HD (Age 65+)	€ 1,144,477,702	64,925,515	€ 152,376,407	-1,414	Dominated	-
UCL	rVE=23.5%	LCL	rVE=-2.5%	Current Strategy	€ 958,080,674	64,924,575	--	--	Ref	-
				aQIV (Age 65+)	€ 984,390,792	64,928,505	€ 26,310,118	3,930	€ 6,695	Current Strategy
				QIV-HD (Age 65+)	€ 1,128,445,290	64,928,807	€ 144,054,498	302	€ 477,483	aQIV (Age 65+)
UCL	rVE=23.5%	Base	rVE=3.2%	Current Strategy	€ 958,080,674	64,924,575	--	--	Ref	-
				aQIV (Age 65+)	€ 984,390,792	64,928,505	€ 26,310,118	3,930	€ 6,695	Current Strategy
				QIV-HD (Age 65+)	€ 1,131,954,940	64,928,094	€ 147,564,148	-411	Dominated	-
UCL	rVE=23.5%	UCL	rVE=8.9%	Current Strategy	€ 958,080,674	64,924,575	--	--	Ref	-
				aQIV (Age 65+)	€ 984,390,792	64,928,505	€ 26,310,118	3,930	€ 6,695	Current Strategy
				QIV-HD (Age 65+)	€ 1,135,927,439	64,927,282	€ 151,536,647	-1,223	Dominated	-

Figure S3. Cost-effectiveness acceptability curve showing the probability that aQIV and QIV-HD are cost-effective compared to the current QIVe in Germany over a range of willingness-to-pay values.

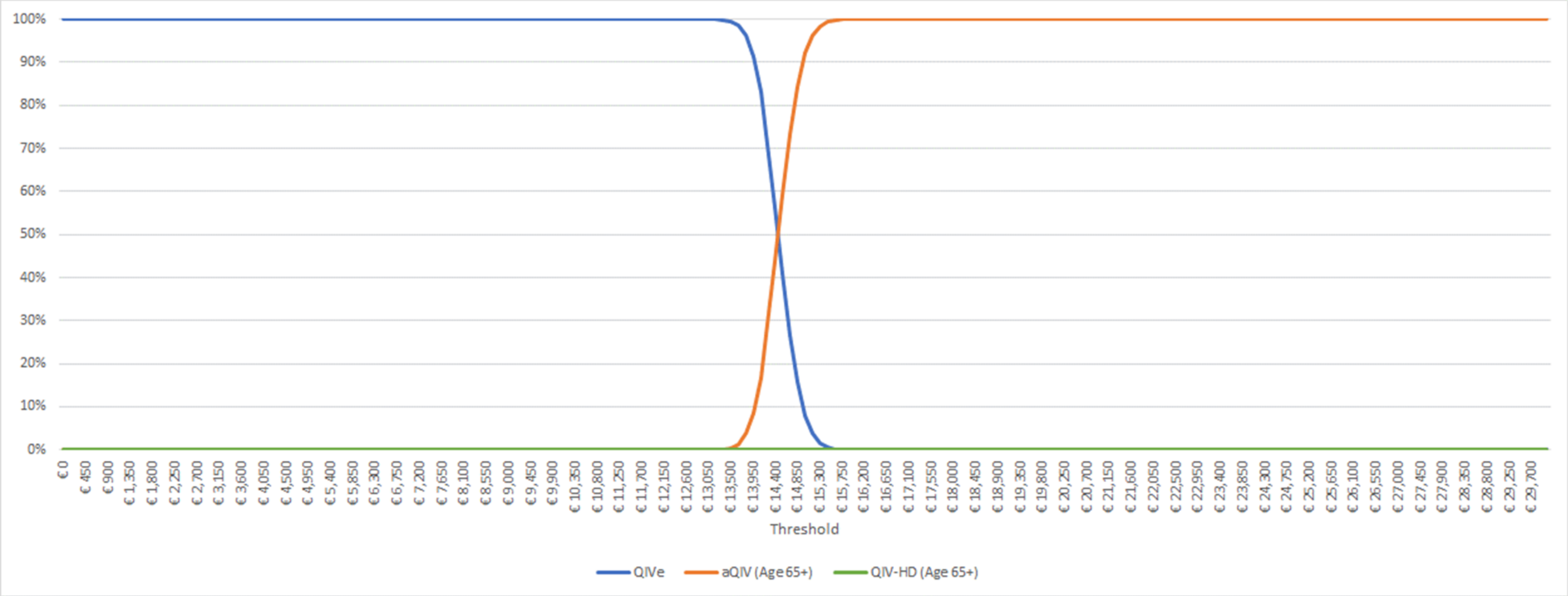

Figure S4. Scatterplot of incremental costs and QALYs from the PSA simulation comparing aQIV to the current QIVe in Germany.

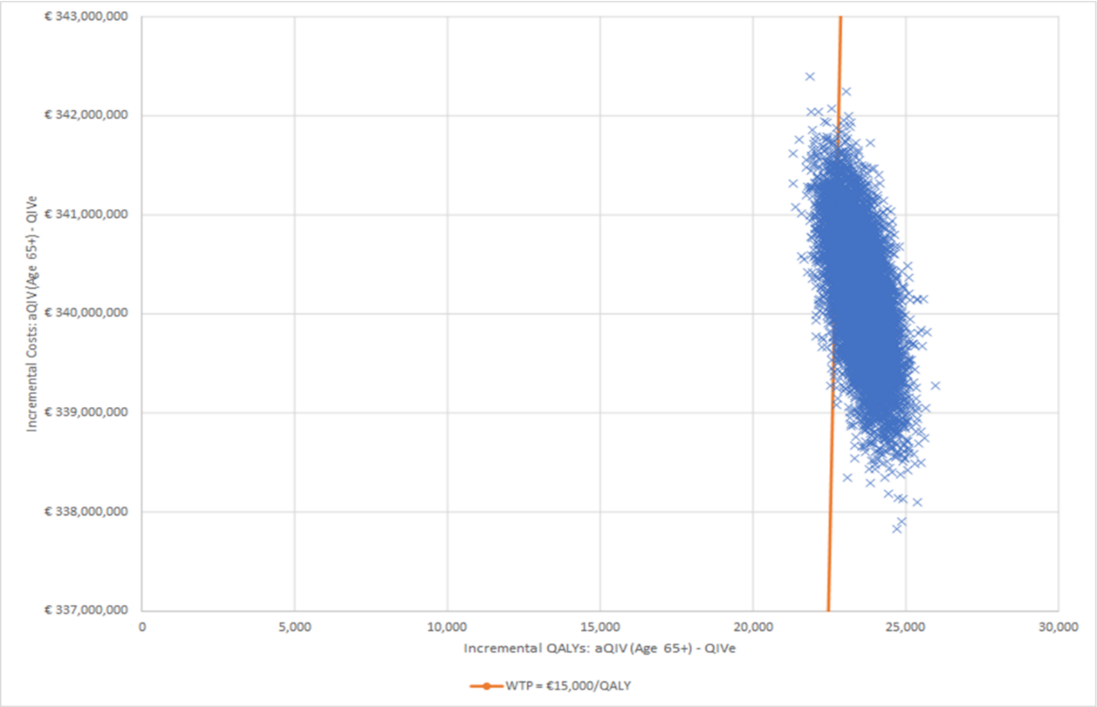

Figure S5. Scatterplot of incremental costs and QALYs from the PSA simulation comparing QIV-HD to aQIV in Germany.

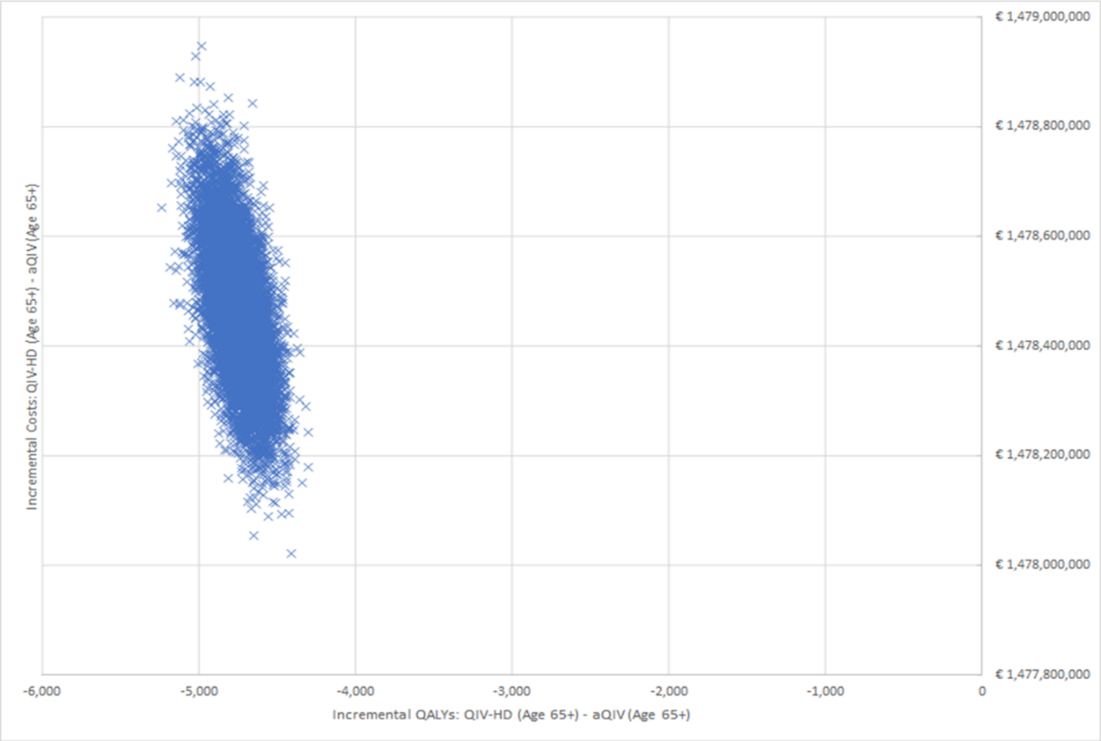

Supplement: Supplementary file 1 [file vaccines-10-01386-s001.zip › vaccines-1809754-supplementary.pdf]
